# Salivary proteomics of canine oral tumors using MALDI-TOF mass spectrometry and LC-tandem mass spectrometry

**DOI:** 10.1371/journal.pone.0219390

**Published:** 2019-07-18

**Authors:** Sekkarin Ploypetch, Sittiruk Roytrakul, Janthima Jaresitthikunchai, Narumon Phaonakrop, Sucheewin Krobthong, Gunnaporn Suriyaphol

**Affiliations:** 1 Biochemistry Unit, Department of Physiology, Faculty of Veterinary Science, Chulalongkorn University, Bangkok, Thailand; 2 Companion Animal Cancer Research Unit, Faculty of Veterinary Science, Chulalongkorn University, Bangkok, Thailand; 3 Proteomics Research Laboratory, Genome Institute, National Center for Genetic Engineering and Biotechnology, National Science and Technology Development Agency, Pathum Thani, Thailand; Virginia Commonwealth University, UNITED STATES

## Abstract

Canine oral tumors are relatively common neoplasms in dogs. For disease monitoring and early diagnosis, salivary biomarkers are appropriate because saliva collection is non-invasive and requires no professional skills. In the era of omics, matrix-assisted laser desorption/ionization with time-of-flight mass spectrometry (MALDI-TOF MS) coupled with liquid chromatography-tandem MS (LC-MS/MS) are suitable to identify potential disease-associated peptides and proteins. The present study aimed to use MALDI-TOF MS and LC-MS/MS to search for particular peptide mass fingerprints (PMFs) and conceivable biomarkers in saliva of dogs with early- and late-stage oral melanoma (EOM and LOM, respectively), oral squamous cell carcinoma (OSCC), benign oral tumors (BN), and periodontitis and healthy controls (CP). Pooled saliva samples in each group were used to be representative of population change. Unique PMFs were obtained and specific peptide fragments were sequenced by LC-MS/MS and BLAST-searched with mammalian protein databases. Seven peptide fragments appeared in the tumor groups (EOM, LOM, OSCC and BN) at 1096, 1208, 1322, 1794, 1864, 2354 and 2483 Da, two peptide fragments appeared in the LOM and OSCC groups at 2450 and 3492 Da, and in the CP controls at 2544 and 3026 Da. Also, protein–chemotherapy drug interaction networks were exhibited. Using western blot analysis, the expression of sentrin-specific protease 7 (SENP7), a peptide fragment at 1096 Da, in OSCC was significantly increased, as was the expression of TLR4, a peptide fragment at 3492 Da, in LOM and OSCC, compared with the CP group. The expression of nuclear factor kappa B (NF-κB), a TLR4 partner, was notably increased in OSCC compared with CP, BN and EOM. The expression was also enhanced in LOM compared with EOM. Expressed protein sequences from western blots were verified by LC-MS/MS. Western blots were then performed with individual samples in each group. The results showed the elevated expression of TLR4 in LOM and OSCC, compared with that in CP and BN, the increased expression of NF-κB in LOM and OSCC, compared with CP and in LOM compared with BN, and the enhanced expression of SENP7 in LOM and OSCC, compared with that in CP and BN. In conclusion, discrete clusters of EOM, LOM, OSCC, BN and CP groups and potential protein candidates associated with the diseases were demonstrated by salivary proteomics. Western blot analysis verified SENP7, TLR4 and NF-κB as potential salivary biomarkers of canine oral tumors.

## Introduction

Tumors in the oral cavity and gastrointestinal system account for 18% of tumors in dogs and tumors in the oral cavity account for 46% of canine head and neck cancers [1, 2]. As the oral cavity is not routinely examined by owners or veterinarians, oral cancers are usually detected at a late clinical stage (stages III and IV), based on the World Health Organization (WHO) clinical staging system for tumors of the oral cavity [3]. As defined by their primary sizes and metastatic profile, the tumor, node and metastasis (TNM) stages comprise stage I (a <2 cm diameter tumor), stage II (a 2 to <4 cm diameter tumor), stage III (a ≥4 cm tumor and/or lymph node metastasis) and stage IV (a tumor with distant metastasis). Surgery resection is the primary option for canine oral tumors. The combination of surgery and chemotherapy drugs is considered for late-stage cancer. The common anti-cancer drugs used are carboplatin, a derivative of the anti-cancer drug cisplatin, doxorubicin (also called adriamycin), cyclophosphamide and piroxicam. The last two drugs are widely used in metronomic chemotherapy [4–6]. Dogs with a late clinical stage have a high mortality rate [7]. Hence, early diagnosis and screening are important for successful treatment. The gold standard for oral tumor diagnosis is a tissue biopsy for histopathological examination; however, it is an invasive technique and impractical for oral cancer screening. Salivary biomarkers are suitable for early detection or monitoring of oral tumors because saliva contacts directly with an oral mass, and saliva collection is non-invasive and easy to perform. In order to discover novel salivary proteins for human oral tumors, a proteomic approach has been performed [8–11]. In healthy dogs, salivary proteomic analysis as well as the comparison of canine salivary proteomics with that of healthy humans have recently been reported [12–15]. However, the study of salivary proteomics of dogs with oral diseases has not been demonstrated. The present study aimed to explore novel peptide mass fingerprints (PMFs), clusters, and conceivable biomarkers in saliva of dogs with early- and late-stage oral melanoma (EOM and LOM, respectively), oral squamous cell carcinoma (OSCC), benign oral tumors (BN), periodontitis and healthy controls, using matrix-assisted laser desorption/ionization with time-of-flight mass spectrometry (MALDI-TOF MS), coupled with liquid chromatography-tandem mass spectrometry (LC-MS/MS). The associations of disease-perturbed proteins with chemotherapy drugs, cisplatin, cyclophosphamide, piroxicam and doxorubicin, were exhibited. The candidate protein expressions were verified by western blot analysis. Our study demonstrated candidate salivary biomarkers of canine oral tumors that might help diagnosis and treatment plan of the diseases.

## Materials and methods

### Animals

Saliva samples were collected from patients with oral tumors scheduled for surgical excision at the Small Animal Teaching Hospital, Faculty of Veterinary Science, Chulalongkorn University and private animal hospitals, including 5 EOM, 24 LOM, 10 OSCC and 11 BN, respectively (age range 7–14 years). OM was classified as early- or late-clinical stages, according to the TNM staging system of the WHO [16]. Inclusion criteria included the presence of benign oral tumors, OM and OSCC, diagnosed without previous treatment either chemotherapy or radiotherapy. The staging of OM and OSCC were determined according to the WHO [17]. Dogs were examined for an oral, regional lymph node, and physical condition; moreover, the regional lymph nodes were required for cytological examination to rule out metastasis. Skull-to-abdomen radiography was evaluated by a Brivo DR-F digital X-ray system (GE Healthcare, Chicago, IL) or Optima CT660 CT-scanner (GE Healthcare). OM and OSCC metastasis to abdominal organs was checked by ultrasonography. Seven samples were gathered from dogs with normal oral health with normal blood profiles and no history or clinical signs of oral cavity or cancerous problems (age range 7–8 years). For a chronic periodontitis group, 5 dogs demonstrated gingivitis, dental tartar and/or periodontal attachment loss (age range 7–13 years). The samples were obtained with the consent of owners and sample collection protocol was approved by the Chulalongkorn University Animal Care and Use Committee (CU-ACUC), Thailand (Approval number 1631042).

### Sample preparation

To collect saliva, dogs were fasted for at least 1 h before saliva collection and mouths were cleaned with 0.9% sterile normal saline solution [10]. Saliva was collected on the day of surgery without mechanical and chemical stimulation. Whole saliva (0.5–1.0 mL) was collected for 5–10 min using a sterile cotton swab. Samples were centrifuged at 2600 x*g* for 15 min at 4 °C [18]. Approximate 200 μL of supernatant was mixed with Halt protease inhibitor cocktail (Thermo Fisher Scientific, Waltham, MA) and kept at -20 °C until analysis. Total protein concentrations from salivary supernatants were evaluated by Lowry’s assay at 690 nm, using bovine serum albumin as a standard [19].

### Analysis of salivary peptides by MALDI-TOF MS

The salivary protein sample of each dog was prepared with 0.1% trifluoroacetic acid (TFA) to the final concentration of 0.2 μg/μL. Samples were mixed with MALDI matrix solution, consisting of 10 mg/ml α-cyano-4-hydroxycinnamic acid in 100% acetonitrile (ACN) and 5% trifluoroacetic acid, at the ratio of 1:1, and directly applied onto the MTP384 target plate (Bruker Daltonics, Billercia, MA) and air dried. Eight replicates were performed to prevent sample preparation bias. Mass spectra were obtained with an Ultraflex III TOF/TOF (Bruker Daltonics) in a linear positive mode with a mass range of 1000–20000 Da. External calibrations were performed using a ProteoMass Peptide & Protein MALDI-MS Calibration Kit (Sigma Aldrich, St. Louis, MO) that consists of human angiotensin II (m/z 1046), P14R (m/z 1533), human adrenocorticotropic hormone fragment 18–39 (m/z 2465), bovine insulin oxidized B chain (m/z 3465), bovine insulin (m/z 5731), and cytochrome *c* (m/z 12362). Saliva peptide mass spectra were determined by flexAnalysis 3.3 software (Bruker Daltonics). Peptide mass spectral peaks were analyzed using Quick Classifier (QC)/ Different Average, Supervised Neural Network (SNN), Anderson-Darling (AD), t-test/ANOVA (TTA), Wilcoxon/Kruskal-Wallis (W/KW) and the Genetic Algorithm (GA) statistical algorithms incorporated in the ClinProTools v. 3.0 software (Bruker Daltonics) to reveal the uniformity and homogeneity of the sample group as PMF, pseudo-gel view and principal component analysis (PCA) [11, 20, 21]. A dendrogram of each dog was constructed, using ClinProTools v. 3.0.

According to dendrograms and PCA plots, four from eight replicates were selected and pooled. Thirty-two replicates of pooled samples were applied twice to MALDI-TOF MS as mentioned above. The recognition capability and cross-validation values of more than 90% exhibited the reliability of the peak selection [22]. ClinProTools v. 3.0 was used to analyze intensity values. Results with p<0.05 were considered significant and peaks were then selected to be analyzed by LC MS/MS.

### Peptide and protein identification by LC-MS/MS

With the limitation of the LC MS/MS, salivary peptide samples at 1000–4000 Da were selected by ClinProTools v. 3.0 and purified using C18 ZipTip (MilliporeSigma, Burlington, MA). Each peptide was diluted in ACN for 51 different dilution ratios equally spaced in the range 0–100%.

Amino acid sequences of gradient-eluted peptides were identified by reversed-phase high performance liquid chromatography and a PTM Discovery System (Bruker Daltonics) coupled to an UltiMate 3000 LC System (Thermo Fisher Scientific, Waltham, MA). Peptides were separated on a nanocolumn (PepSwift monolithic column 100 μm diameter × 50 mm length). The nanoLC system was connected to an electrospray ionization in the positive ion mode and quadrapole ion-trap MS (Bruker Daltonics). Eluent A and eluent B solutions were prepared from a 0.1% formic acid dilution and from 50% ACN in water containing 0.1% formic acid, respectively. Peptide separation was achieved with a 4–70% linear gradient of eluent B at a flow rate of 1000 nL/min for 7.5 min. A regeneration step and an equilibration step were performed with 90% and 4% eluent B, respectively, for 20 min per run. Peptide fragment mass spectra were acquired in the data-dependent AutoMS mode with a scan range of 400–1500 m/z, 3 averages, and up to 5 precursor ions, selected from the MS scan at 200–2800 m/z.

The results of LC MS/MS were converted into an mzXML file by CompassXport software (Bruker Daltonics). DeCyder MS Differential Analysis software (Amersham Biosciences, Little Chalfont, UK) was used for protein quantification [23, 24] The PepDetect module was used in MS mode for automated peptide detection, charge state assignments, and peptide ion signal intensities. The proteins were identified from MS/MS peptide mass values using Mascot software (Matrix Science, London, UK) [25]. The data were searched against the NCBI mammal database for protein identification. Proteins were identified from one or more peptides with an individual MASCOT score corresponding to p<0.05. The information about particular proteins and detailed analysis of the protein sequences were used in the annotation of UniProtKB/Swiss-Prot entries [26]. The relationship of candidate proteins and chemotherapy drugs were performed by the Stitch program, version 5.0 [27].

### Validation of MS results by western blot analysis

Pooled saliva samples of 5 μg for SENP7 and TLR4 and of 12 μg for NF-κB were mixed with loading dye [0.5 M dithiothreitol, 10% (w/v) SDS, 0.4 M Tris-HCl, pH 6.8, and 50% (v/v) glycerol]. Samples were heated at 85 °C for 10 min prior to loading on pre-cast NuPAGE 4–12% (w/v) Bis-Tris 1.0-mm minigel (Thermo Fisher Scientific), using RunBlue MES Run Buffer (Expedeon, Heidelberg, Germany) in an XCell SureLock Mini-Cell electrophoresis system (Thermo Fisher Scientific) at 200 V for 90 min. PageRuler prestained protein ladder (molecular weight range 10–180 kDa) (Thermo Fisher Scientific) was used. Subsequently, proteins were transferred to Trans-Blot Turbo mini-sized nitrocellulose membranes (Bio-Rad Laboratories, Hercules, CA) at 25 V for 14 min using Trans-Blot Turbo 5× transfer buffer (Bio-Rad Laboratories). A Pierce Reversible Protein Stain Kit for Nitrocellulose Membranes (Thermo Fisher Scientific) was used to detect total proteins in each well according to the manufacturer’s instructions. Nonspecific binding was blocked with 5% bovine serum albumin (GoldBio, St Louis MO) in Tris-buffered saline containing 0.1% Tween 20 (TBST) overnight. After washing with TBST, primary antibodies, 1:1000 mouse monoclonal anti-mouse TLR4 (25) (sc-293072, Santa Cruz Biotechnology, Dallas, TX), 1:1000 mouse monoclonal anti-human SENP7 (E-8) (sc- 373821, Santa Cruz Biotechnology) or 1:1000 mouse monoclonal anti-human NF-κB p65 (sc- 8008, Santa Cruz Biotechnology) were incubated with a membrane at 4 °C overnight. A membrane was washed and subsequently incubated with 1:10,000 horseradish peroxidase-conjugated rabbit anti-mouse antibody (ab6728, Abcam, Cambridge, UK) at 25 °C for 1 h. The proteins of interest were detected using ECL western blotting detection reagents (GE Healthcare). Western blots were imaged with a ChemiDoc Touch Imaging System (Bio-Rad Laboratories). Protein band intensities were analyzed by Image Lab 6.0.1 software (Bio-Rad Laboratories). For western blot normalization, total protein normalization, modified from Aldridge et al. (2008) was used [28]. The ratios of target band intensities to the total proteins in each lane in the first or second half of a membrane were calculated according to the sizes of target proteins. The western blotting was performed in triplicate. Statistical analyses of protein expression data were conducted using GraphPad Prism v. 8.0.1 (GraphPad Software, La Jolla, CA). Western blots were also performed with individual samples in each group after the target protein sequences were confirmed by LC-MS/MS.

### Verification of expressed protein sequences by LC-MS/MS

To confirm SENP7, TLR4 and NF-κB protein identities, Antibodies were removed from nitrocellulose membranes by incubating with Restore Plus Western Blot Stripping Buffer (Thermo Fisher Scientific) at room temperature for 15 min. After washing 4 times with TBST, protein bands were cut and incubated with 10 mM DTT in 10 mM ammonium bicarbonate (Ambic) at room temperature overnight. Samples were then incubated with 10 ng trypsin in 10 mM Ambic at 37 °C for 3 hr and concentrated by the speed-vac (Thermo Fisher Scientific). Fifteen μL of 0.1% formic acid was used to dissolve proteins prior to applying to the LC-MS/MS as mentioned above.

### Statistical analysis

ClinProTools v. 3.0 and MASCOT softwares were used to analyze peak intensities of peptides in MALDI-TOF MS spectra and MASCOT LC-MS/MS scores, respectively. Western blot band intensity ratios were tested for normality and statistical differences were analyzed by ordinary one-way ANOVA with Bonferroni’s multiple comparisons for TLR4 and NF-κB (pooled samples), ordinary one-way ANOVA with Tukey’s multiple comparisons for SENP7 (pooled samples), and Kruskall Wallis with Dunn’s multiple comparisons for TLR4, NF-κB and SENP7 (individual samples). Significance was accepted at the p<0.05 level.

## Results

**Fig 1:**
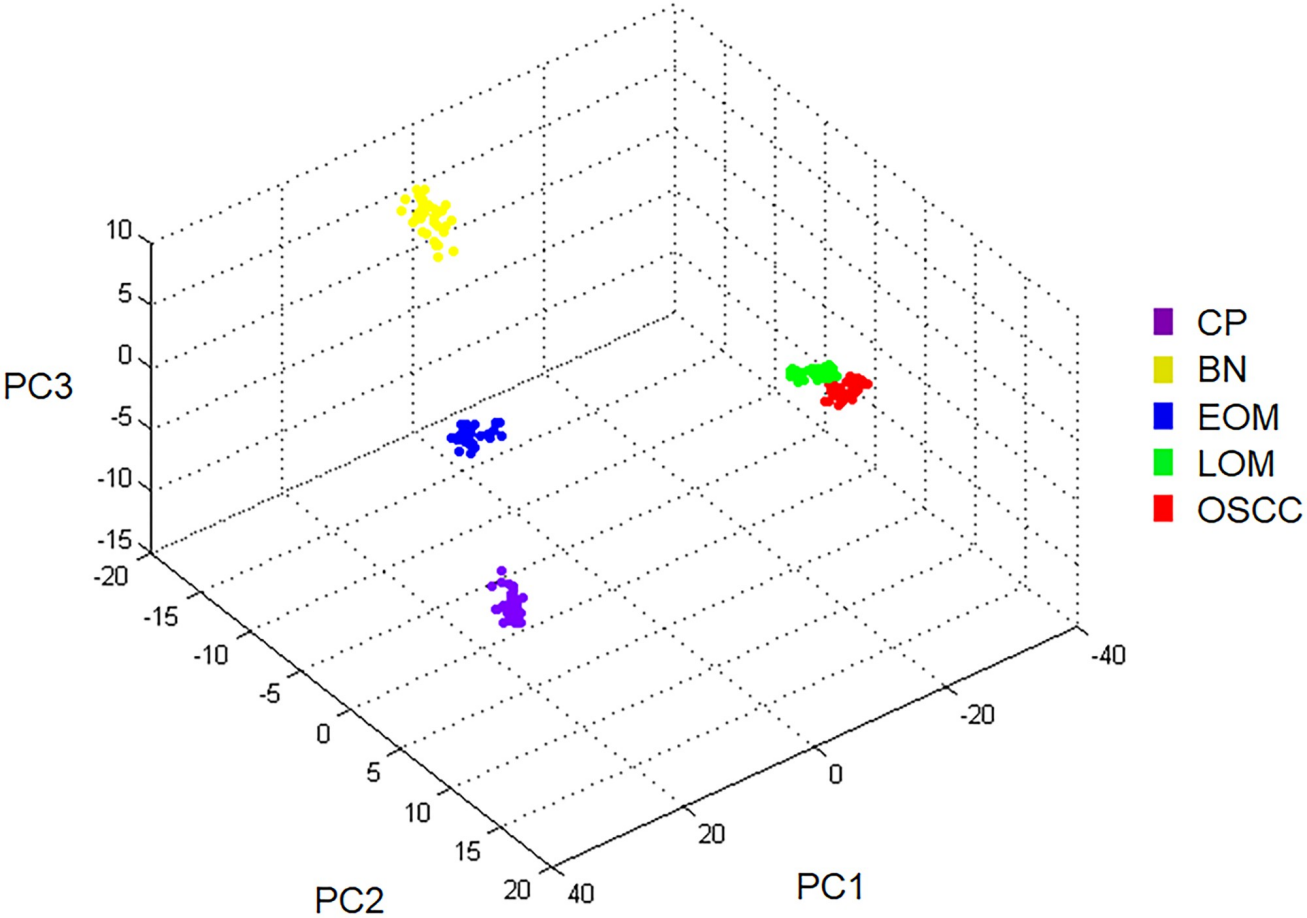
Three-dimensional principal component analysis scatterplot of normal and periodontitis gingiva tissues (CP), benign tumors (BN), early-stage oral melanoma (EOM), late-stage OM (LOM) and oral squamous cell carcinoma (OSCC). Thirty two dots represent replicate in each pooled sample group.

**Fig 2:**
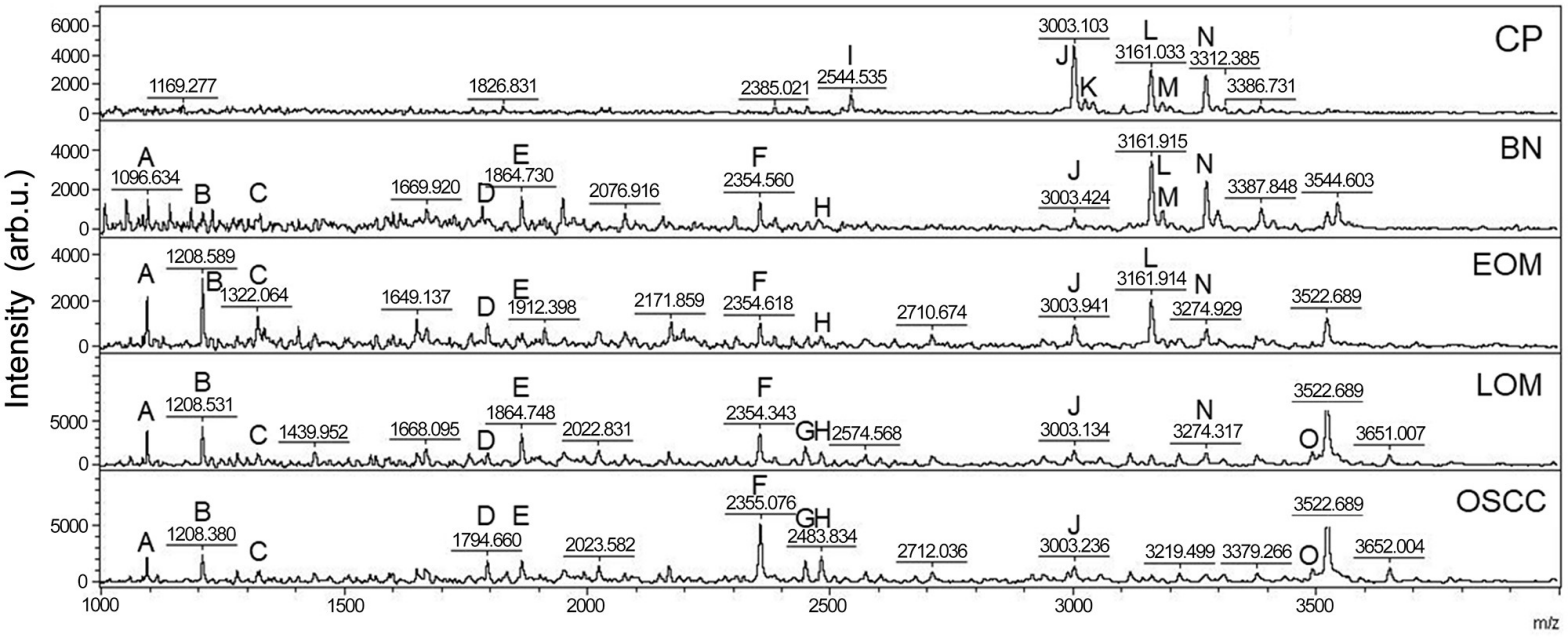
Peptide mass fingerprint (PMF) of normal and periodontitis gingiva tissues (CP), benign tumors (BN), early-stage oral melanoma (EOM), late-stage OM (LOM) and oral squamous cell carcinoma (OSCC) in the range 1000–5000 Da with identified proteins of each mass spectrum. A: SENP7 or KAT2B (1096 Da); B: PPRC1 or RMND1 (1208 Da); C: DTX3L (1322 Da); D: ZNF699) (1794 Da); E: MAP3K15 or ATP6V1E2 (1864 Da); F: PLCL2 (2354 Da); G: TNRC18 (2450 Da); H: COL12A1 (2483 Da); I: ZNF451 (2544 Da); J: protocadherin FAT1 (3003 Da); K: CASPL4A2 (3026 Da); L: centrosomal protein 192 (3161 Da); M: glypican 5 (3184 Da); N: cell-cycle checkpoint protein RAD17 (3274 Da); O: TLR4 (3492 Da). arb. u., arbitrary unit.

**Fig 3:**
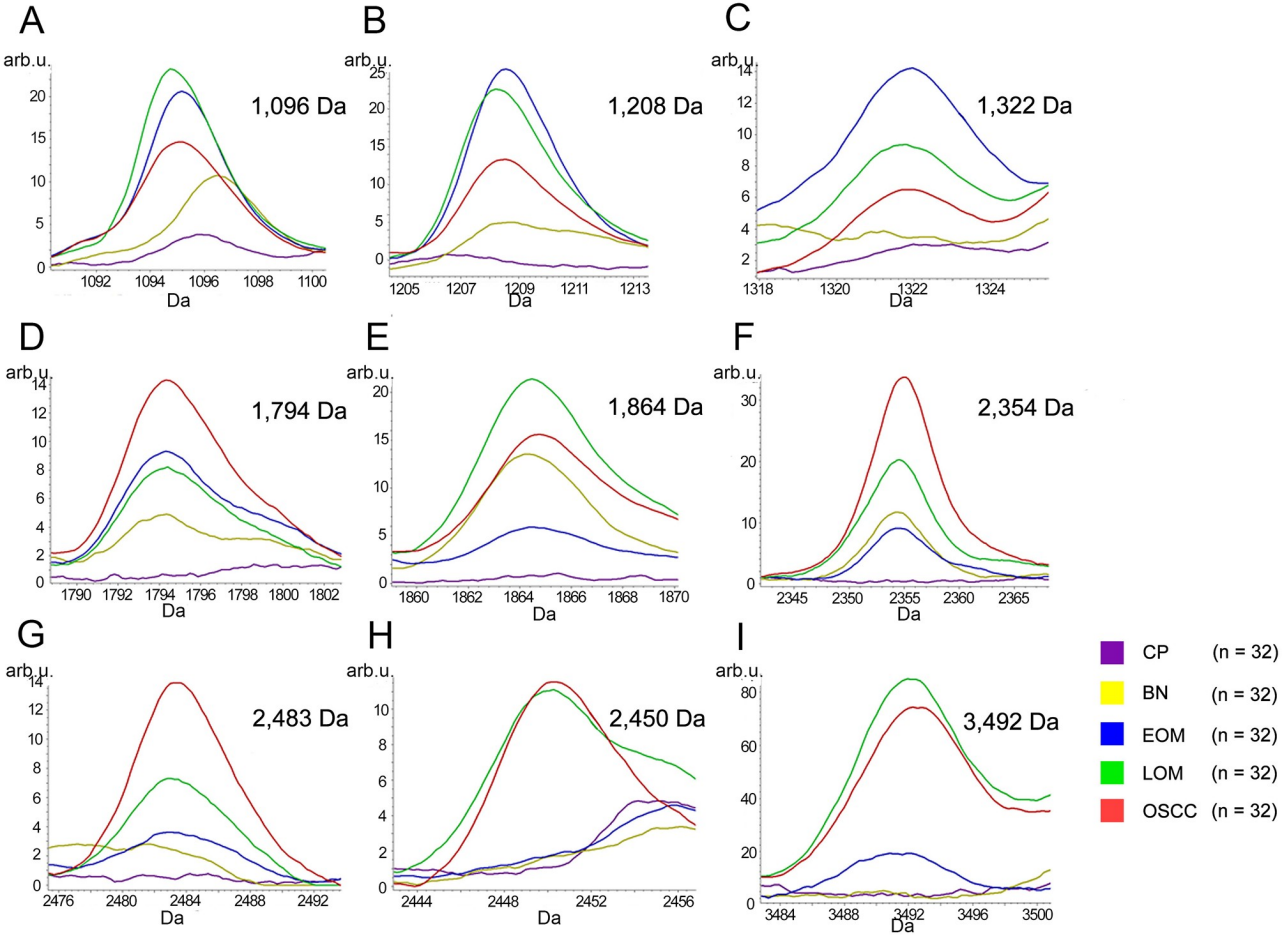
MALDI-TOF intensity profiles of salivary proteins from benign tumors (BN), early-stage oral melanoma (EOM), late-stage OM (LOM) and oral squamous cell carcinoma (OSCC). Percentages of interpretable mass signals are shown: SENP7 or KAT2B at 1096 Da (A); PPRC1 or RMND1 at 1208 Da (B); DTX3L at 1322 Da (C); ZNF699 at 1794 Da (D); MAP3K15 or ATP6V1E2 at 1864 Da (E); PLCL2 at 2354 Da (F); COL12A1 at 2483 Da (G); TNRC18 at 2450 Da (H); TLR4 at 3492 Da (I). arb. u., arbitrary unit.

**Fig 4:**
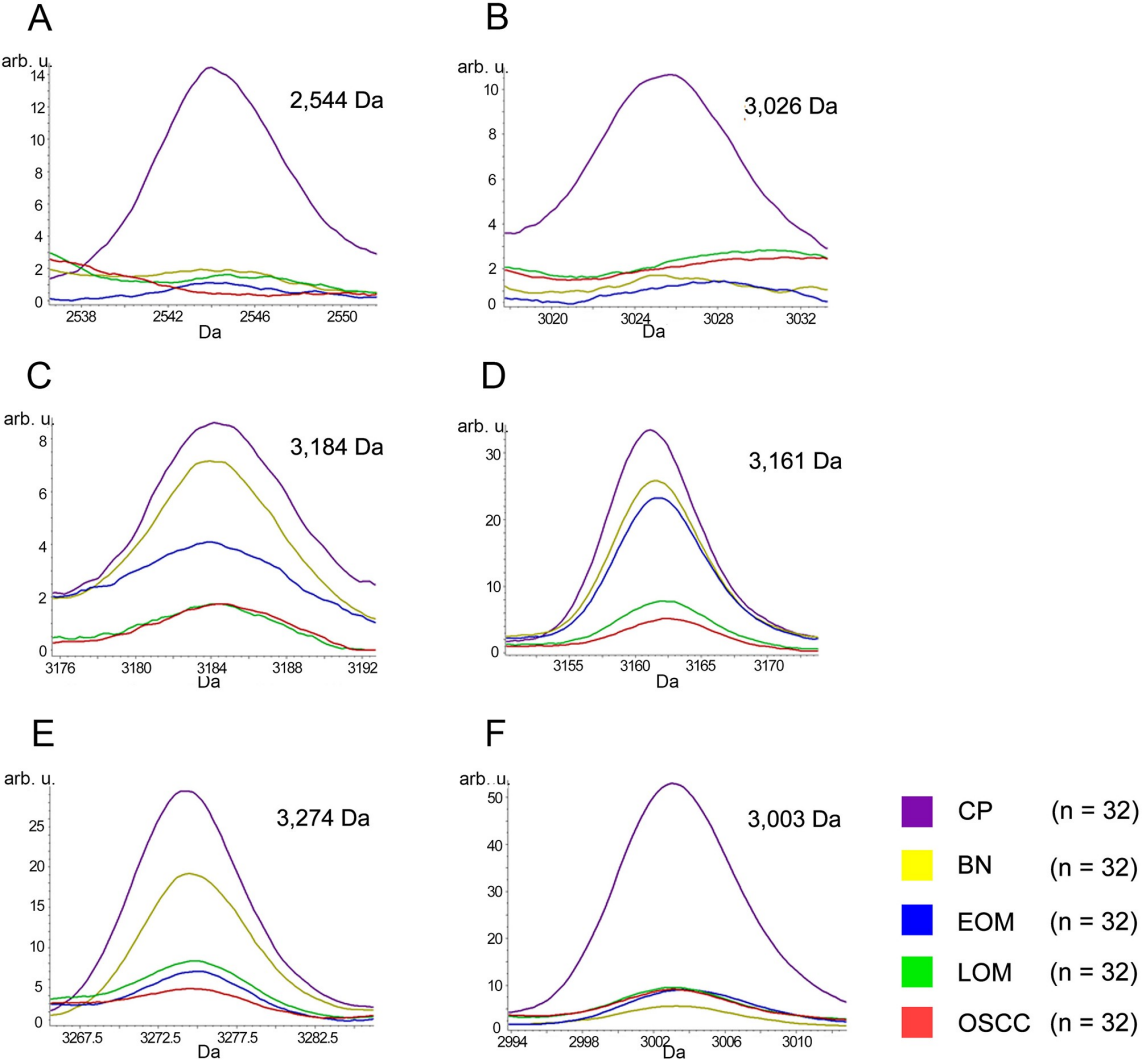
MALDI-TOF intensity profiles of salivary proteins from normal and periodontitis gingiva tissues (CP), benign tumors (BN), early-stage oral melanoma (EOM), late-stage OM (LOM) and oral squamous cell carcinoma (OSCC). Percentages of interpretable mass signals are shown: ZNF451 at 2544 Da (A); CASPL4A2 at 3026 Da (B); glypican 5 at 3184 Da (C); CEP192 at 3161 Da (D); cell-cycle checkpoint protein RAD17 at 3274 Da (E); protocadherin FAT1 at 3003 Da (F). arb. u., arbitrary unit.

**Fig 5:**
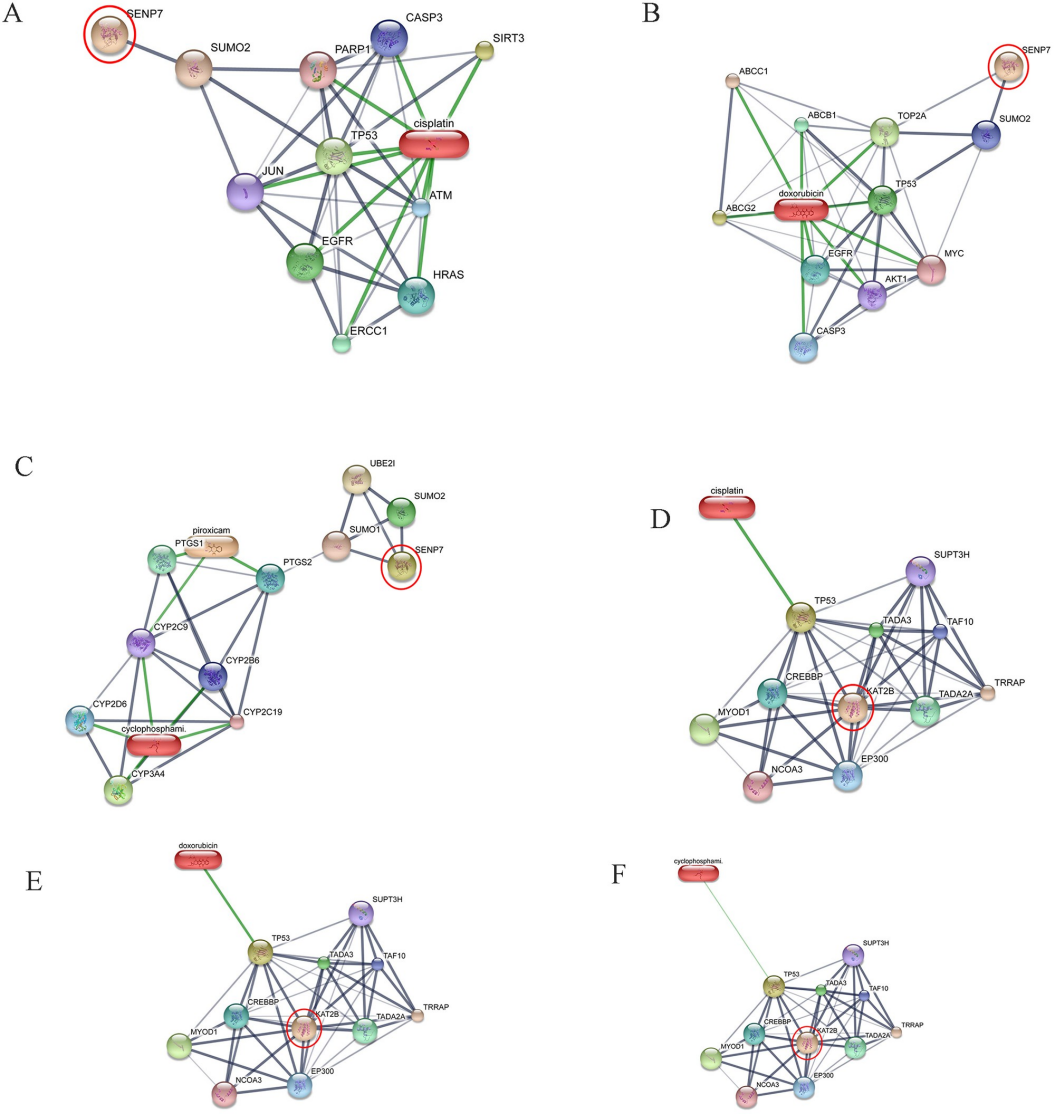
Involvement of sentrin-specific protease 7 (SENP7) and K(lysine) acetyltransferase 2B (KAT2B) in networks of protein–chemotherapy drug interactions, cisplatin, doxorubicin, cyclophosphamide and piroxicam. Interactions of SENP7 with cisplatin (A), SENP7 with doxorubicin **(B)**, SENP7 with cyclophosphamide and piroxicam **(C)**, KAT2B with cisplatin **(D)**, KAT2B with doxorubicin **(E)**, KAT2B with cyclophosphamide and piroxicam **(F)** were exhibited. Red circles: SENP7 and KAT2B. Abbreviations: ATP-binding cassette, sub-family B (MDR/TAP), member 1 (ABCB1), ataxia telangiectasia mutated (ATM), ATP-binding cassette, sub-family C (CFTR/MRP), member 1 (ABCC1), ATP-binding cassette, sub-family G (WHITE), member 2 (ABCG2), v-akt murine thymoma viral oncogene homolog 1 (AKT1), caspase 3 (CASP3), c-jun (JUN), CREB binding protein (CREBBP), cytochrome P450 family 2 subfamily B member 6 (CYP2B6), cytochrome P450 family 2 subfamily C member 9 (CYP2C9), cytochrome P450 family 2 subfamily C member 19 (CYP2C19), cytochrome P450 family 2 subfamily D member 6 (CYP2D6), cytochrome P450 family 3 subfamily A member 4 (CYP3A4), E1A binding protein p300 (EP300), epidermal growth factor receptor (EGFR), v-myc myelocytomatosis viral oncogene homolog (MYC), DNA excision repair protein ERCC-1, endonuclease non-catalytic subunit (ERCC1), Harvey rat sarcoma viral oncogene homolog (HRAS), myogenic differentiation 1 (MYOD1), nuclear receptor coactivator 3 (NCOA3), poly [ADP-ribose] polymerase 1 (PARP-1), prostaglandin-endoperoxide synthase 1 (PTGS1), prostaglandin-endoperoxide synthase 2 (PTGS2), sirtuin 3 (SIRT3), solute carrier family 30 (zinc transporter), member 6 (SLC30A6), suppressor of Ty 3 homolog (S. cerevisiae) (SUPT3H), SMT3 suppressor of mif two 3 homolog 1 (SUMO1), SMT3 suppressor of mif two 3 homolog 2 (SUMO2), transcriptional adaptor 2A (TADA2A), transcriptional adaptor 3 (TADA3), TAF10 RNA polymerase II (TAF10), topoisomerase (DNA) II alpha (TOP2A), transformation/transcription domain-associated protein (TRRAP), tumor protein p53 (TP53), ubiquitin-conjugating enzyme E2I (UBE2I).

**Fig 6:**
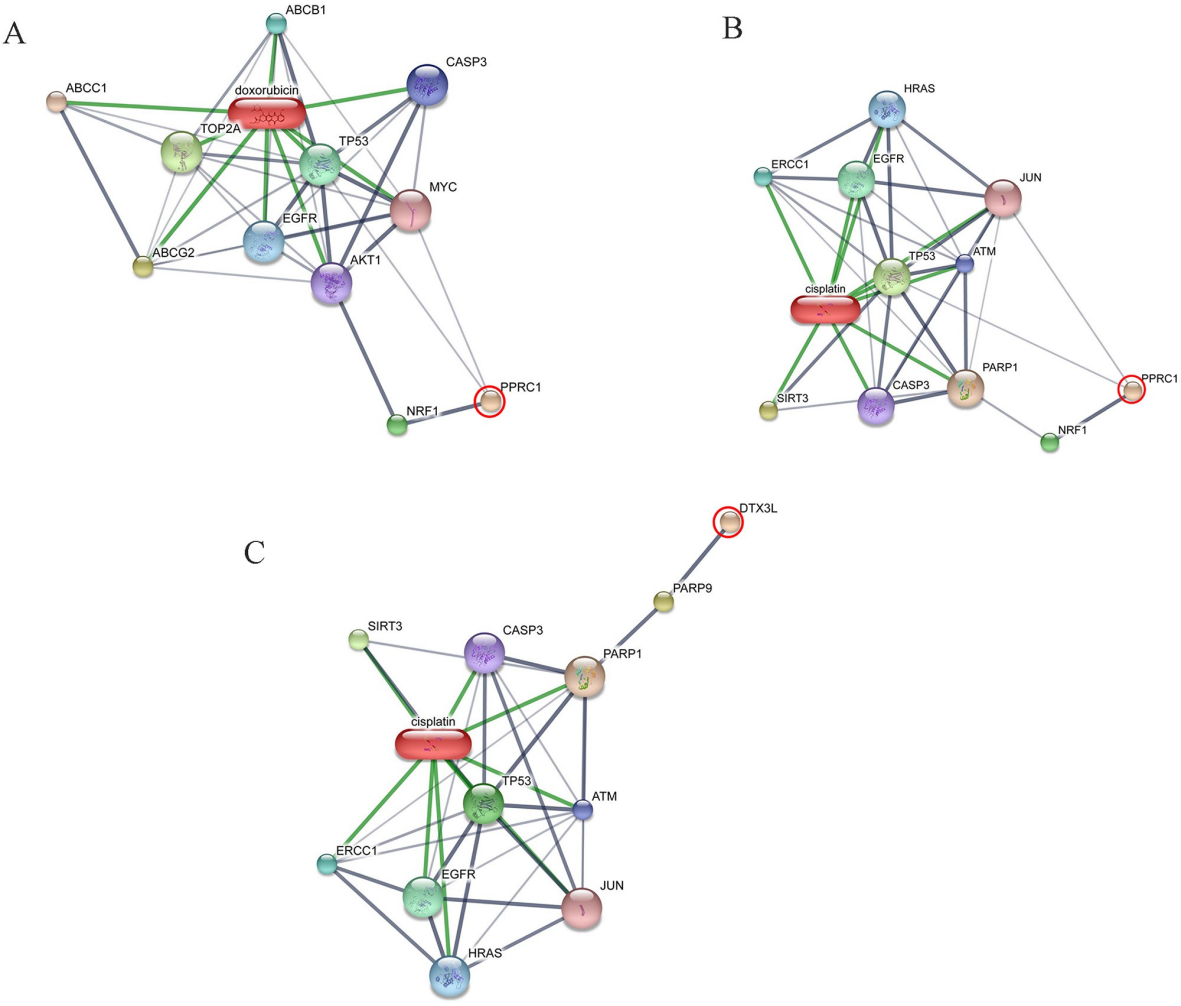
Involvement of peroxisome proliferator-activated receptor gamma, coactivator-related 1 (PPRC1) and deltex 3-like (DTX3L) in networks of protein–chemotherapy drug interactions, cisplatin and doxorubicin. Interactions of PPRC1 with doxorubicin **(A)**, PPRC1 with cisplatin **(B)** and DTX3L with cisplatin **(C)** are shown. Red circles: PPRC1 and DTX3L. Abbreviations: ABCB1, ATP-binding cassette, sub-family B, member 1; ABCC1, ATP-binding cassette, sub-family C, member 1; ABCG2, ATP-binding cassette, sub-family G, member 2; AKT1, v-akt murine thymoma viral oncogene homolog 1; ATM, ataxia telangiectasia mutated; CASP3, caspase 3; EGFR, epidermal growth factor receptor; ERCC1, excision repair cross-complementing rodent repair deficiency, complementation group 1; HRAS, v-Ha-ras Harvey rat sarcoma viral oncogene homolog; JUN, jun proto-oncogene; MYC, v-myc myelocytomatosis viral oncogene homolog; nuclear respiratory factor 1 (NRF1), PARP1, poly (ADP-ribose) polymerase 1; PARP9, poly (ADP-ribose) polymerase family, member 9; SIRT3, sirtuin 3; TOP2A, topoisomerase II alpha; TP53, tumor protein p53.

**Fig 7:**
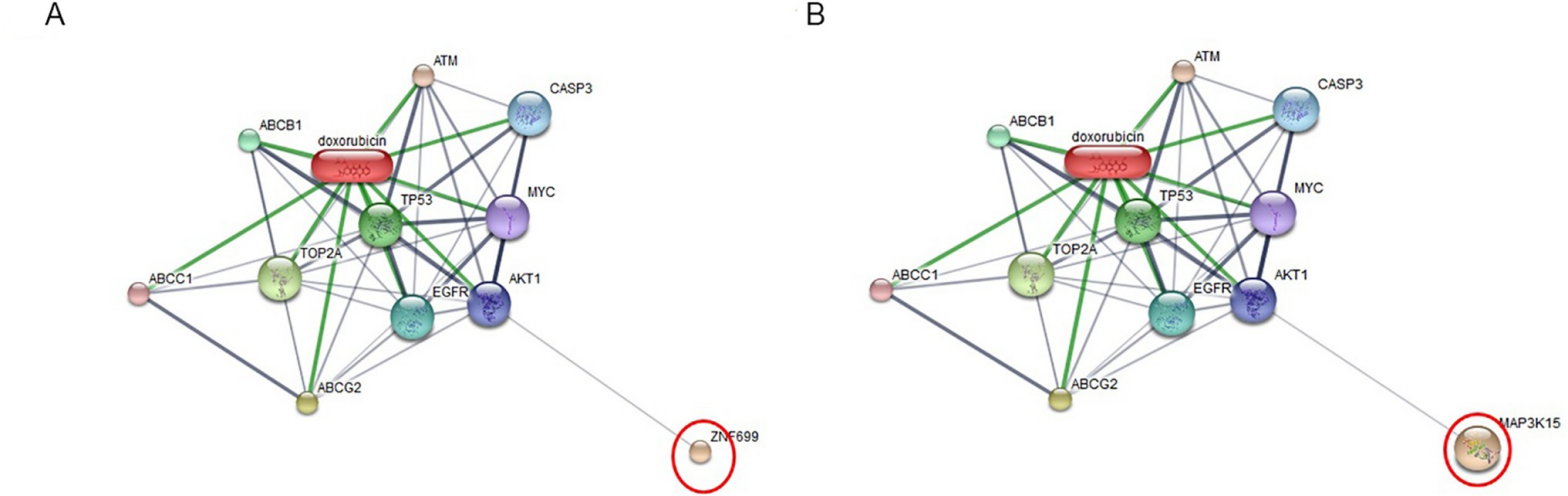
Involvement of zinc finger protein 699 (ZNF699) (A) and mitogen-activated protein kinase kinase kinase 15 (MAP3K15) (B) in networks of protein–chemotherapy drug interactions, doxorubicin. Red circles: ZNF699 and MAP3K15. Abbreviations: ABCB1, ATP-binding cassette, sub-family B, member 1; ABCC1, ATP-binding cassette, sub-family C, member 1; ABCG2, ATP-binding cassette, sub-family G, member 2; AKT1, v-akt murine thymoma viral oncogene homolog 1; ATM, ataxia telangiectasia mutated; CASP3, caspase 3; EGFR, epidermal growth factor receptor; MYC, v-myc myelocytomatosis viral oncogene homolog; TOP2A, topoisomerase II alpha; TP53, tumor protein p53.

**Fig 8:**
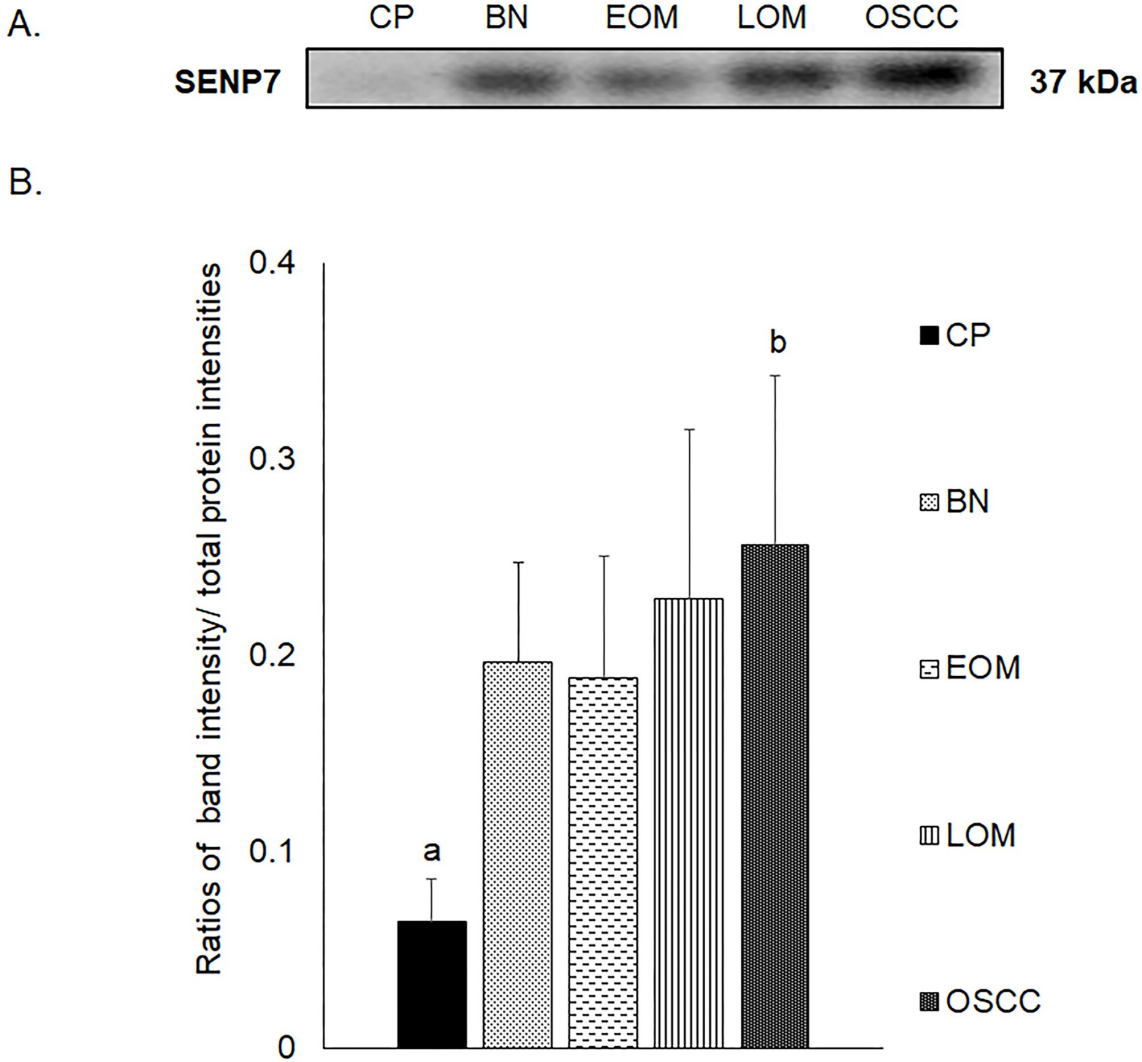
Western blot analysis of salivary sentrin-specific protease 7 (SENP7) of pooled saliva samples from dogs with periodontitis and normal controls (CP), benign oral tumors (BN), early- and late-stage oral melanoma (EOM and LOM, respectively) and oral squamous cell carcinoma (OSCC). Representative western blot for SENP7 at 37 kDa **(A)** and bar graph of ratios of SENP7 protein intensity to total blotted protein intensities in each lane in the second half of a membrane* **(B)**. a–b denote a significant difference at p<0.05.

**Fig 9:**
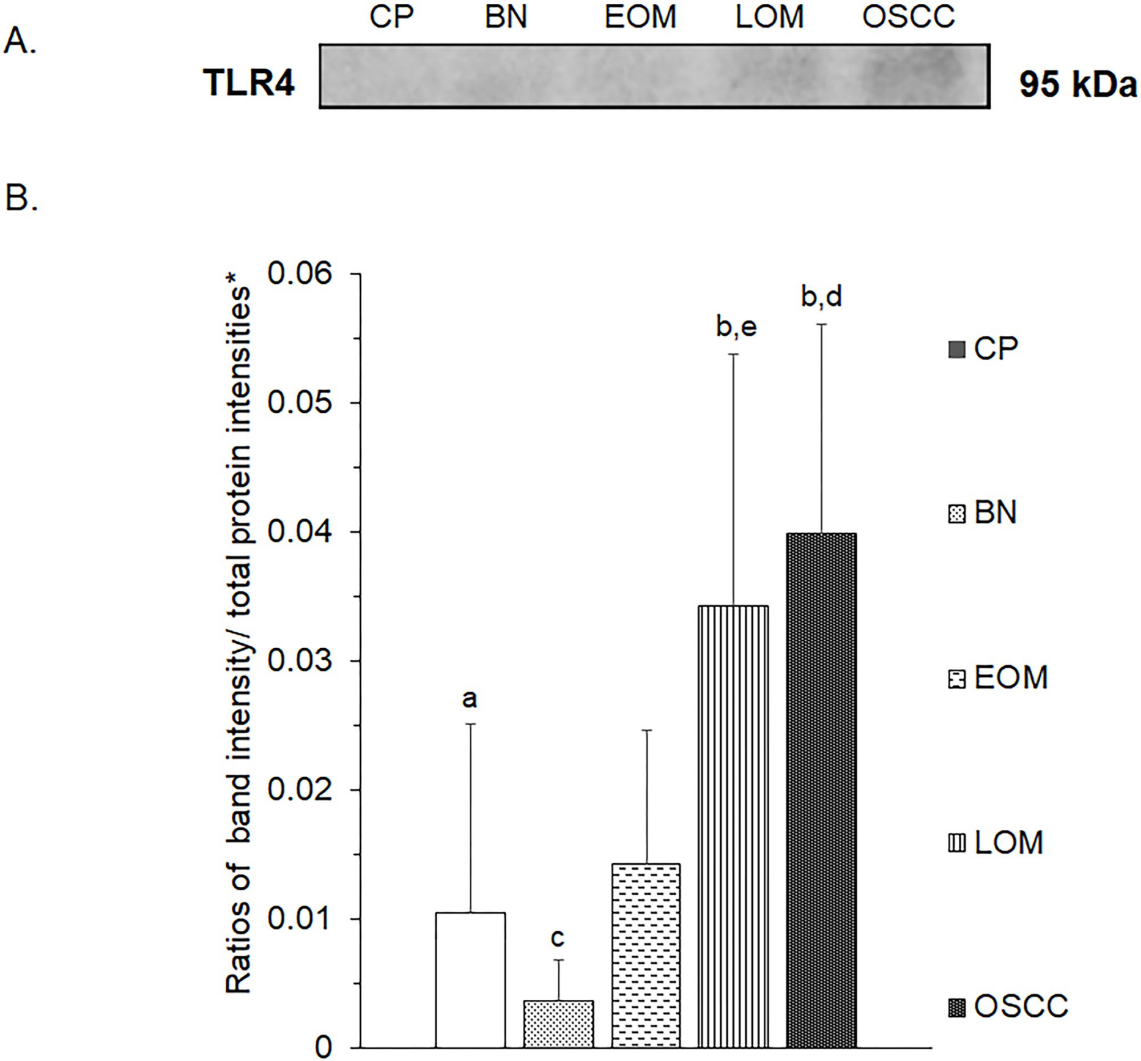
Western blot analysis of salivary toll like receptor 4 (TLR4) of pooled saliva samples from dogs with benign oral tumors (BN), early- and late-stage oral melanoma (EOM and LOM, respectively), oral squamous cell carcinoma (OSCC) and periodontitis and normal controls (CP). Representative western blot for TLR4 at 95 kDa **(A)** and bar graph of ratios of TLR4 protein intensity to total blotted proteins in each lane in the first half of a membrane* **(B)**. a–b denote a significant difference at p<0.05. c–d denote a significant difference at p<0.01.

**Fig 10:**
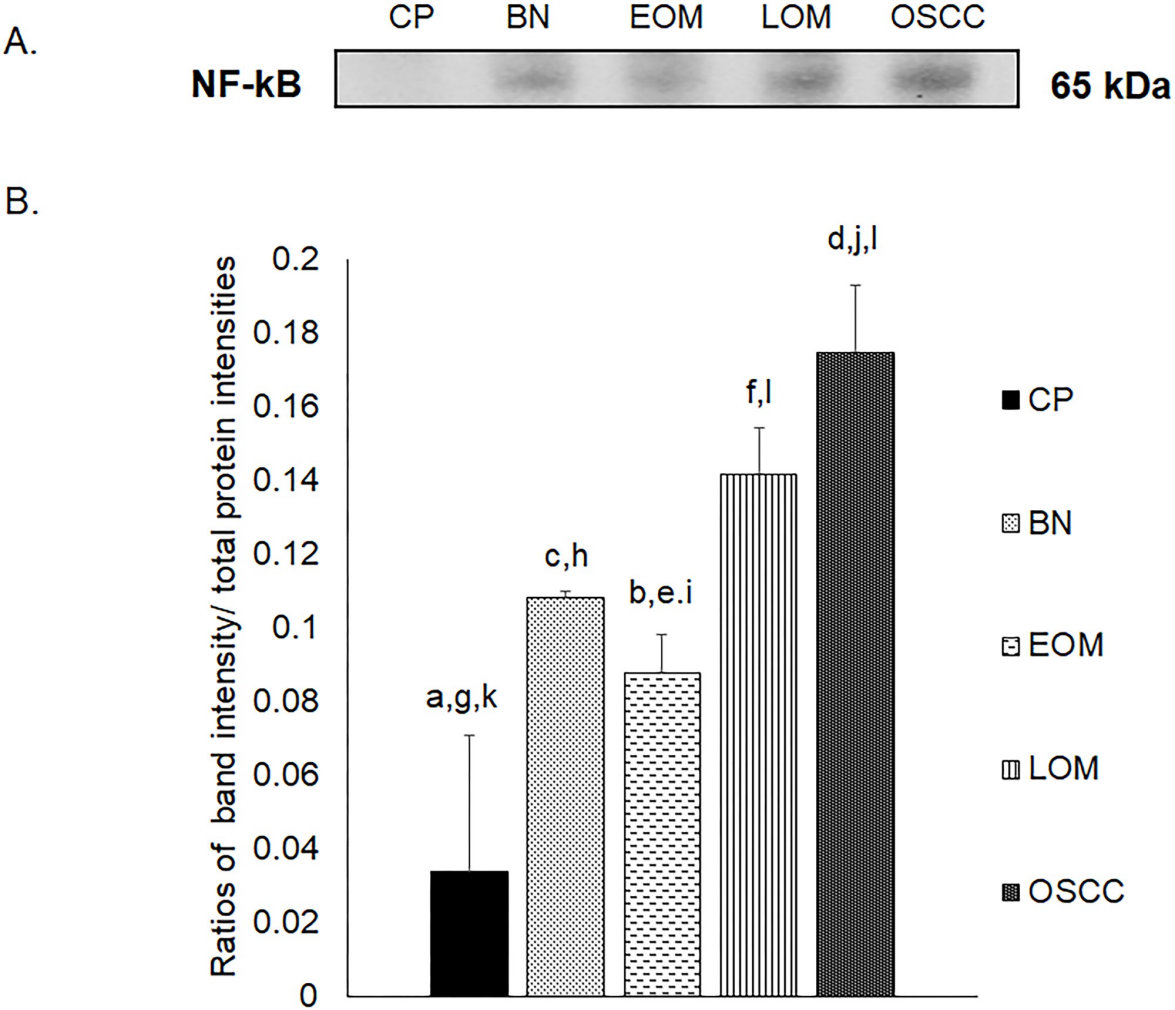
Western blot analysis of nuclear factor kappa B (NF-κB) of pooled saliva samples from dogs with benign oral tumors (BN), early- and late-stage oral melanoma (EOM and LOM, respectively), oral squamous cell carcinoma (OSCC) and periodontitis and normal controls (CP). Representative western blot for NF-κB at 65 kDa **(A)** and bar graph of ratios of NF-κB protein intensity to total blotted proteins in each lane in the first half of a membrane* **(B)**. a–b, c–d and e–f denote a significant difference at p<0.05. g–h and i–j denote a significant difference at p<0.01. k–l denote a significant difference at p<0.001.

**Fig 11:**
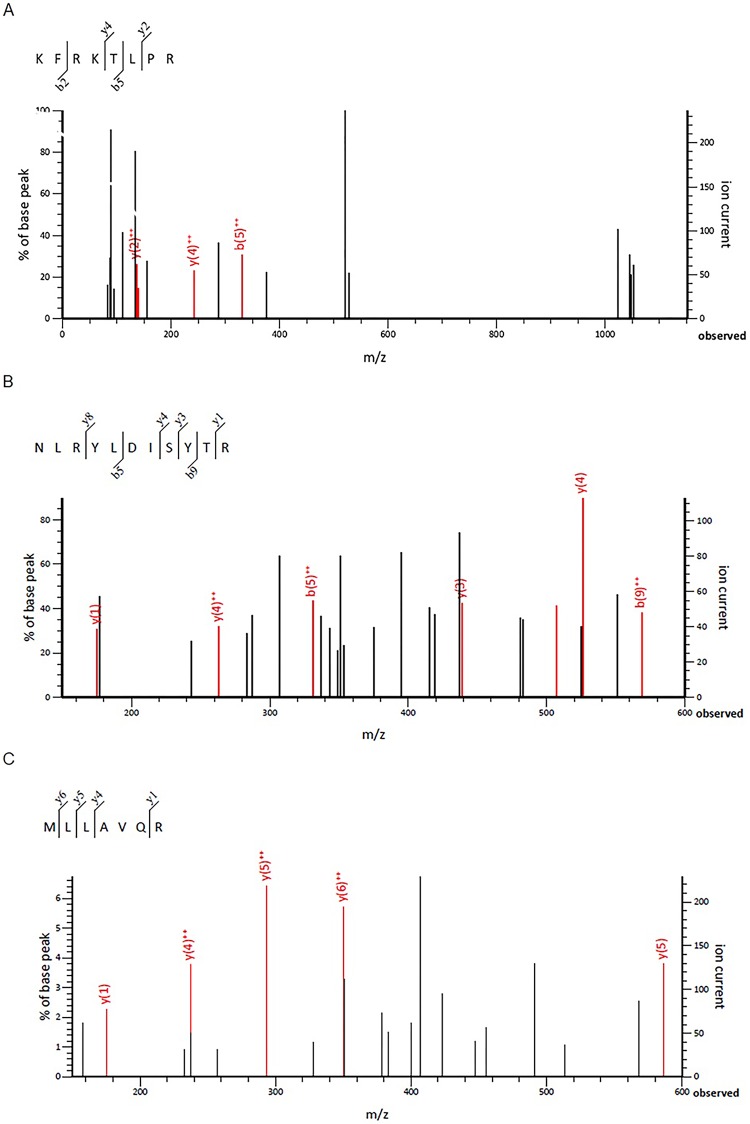
Verification of expressed protein sequences by LC-MS/MS. MS/MS fragmentations of KFRKTLPR found in SENP7 **(A)**, NLRYLDISYTR found in TLR4 **(B)**, and MLLAVQR found in NF-κB **(C)** were shown.

**Fig 12:**
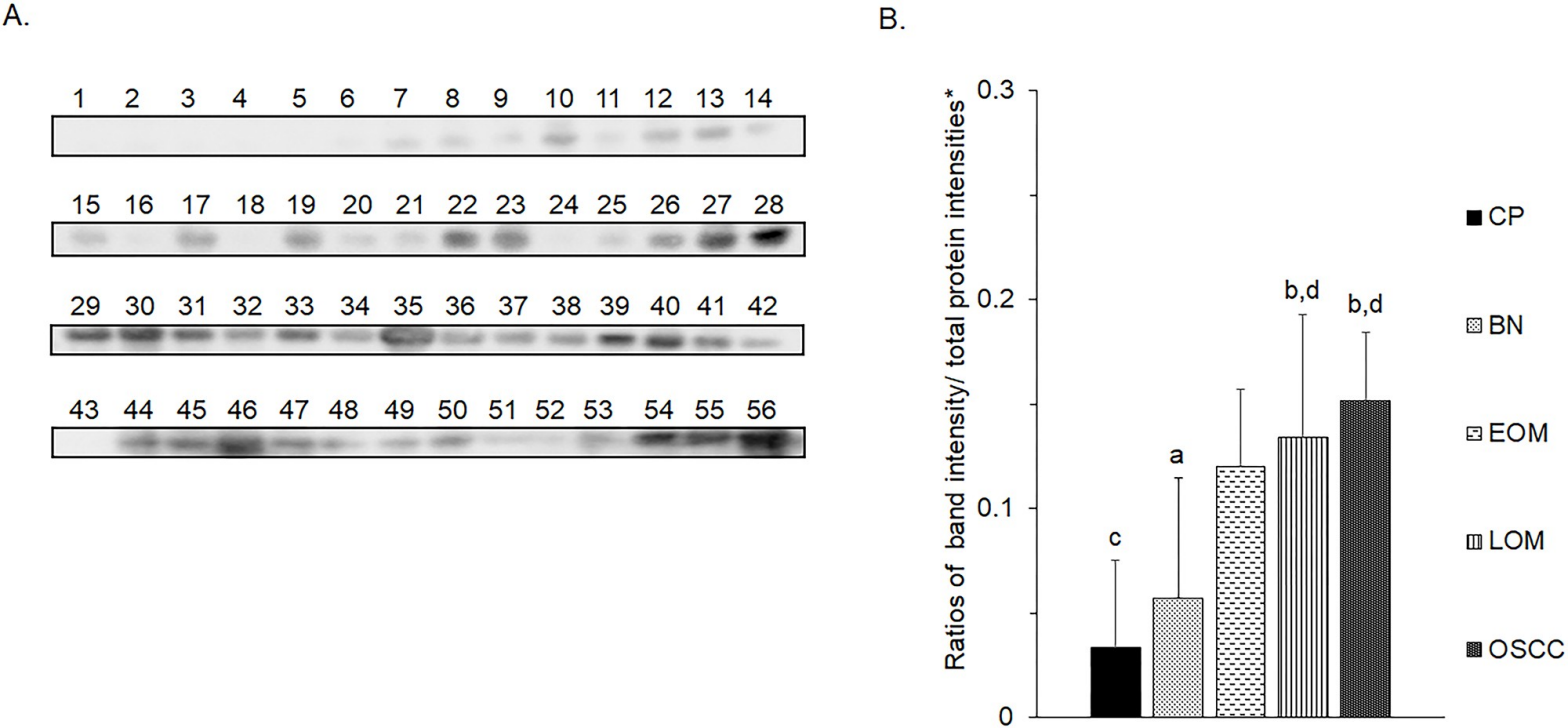
Western blot analysis of salivary sentrin-specific protease 7 (SENP7) of individual saliva samples from dogs with periodontitis and normal controls (CP), benign oral tumors (BN), early- and late-stage oral melanoma (EOM and LOM, respectively) and oral squamous cell carcinoma (OSCC). Representative western blot for SENP7 at 37 kDa **(A)** and bar graph of ratios of SENP7 protein intensity to total blotted protein intensities in each lane in the second half of a membrane* **(B)**. a–b and c-d denote a significant difference at p<0.05 and p<0.01, respectively.

**Fig 13:**
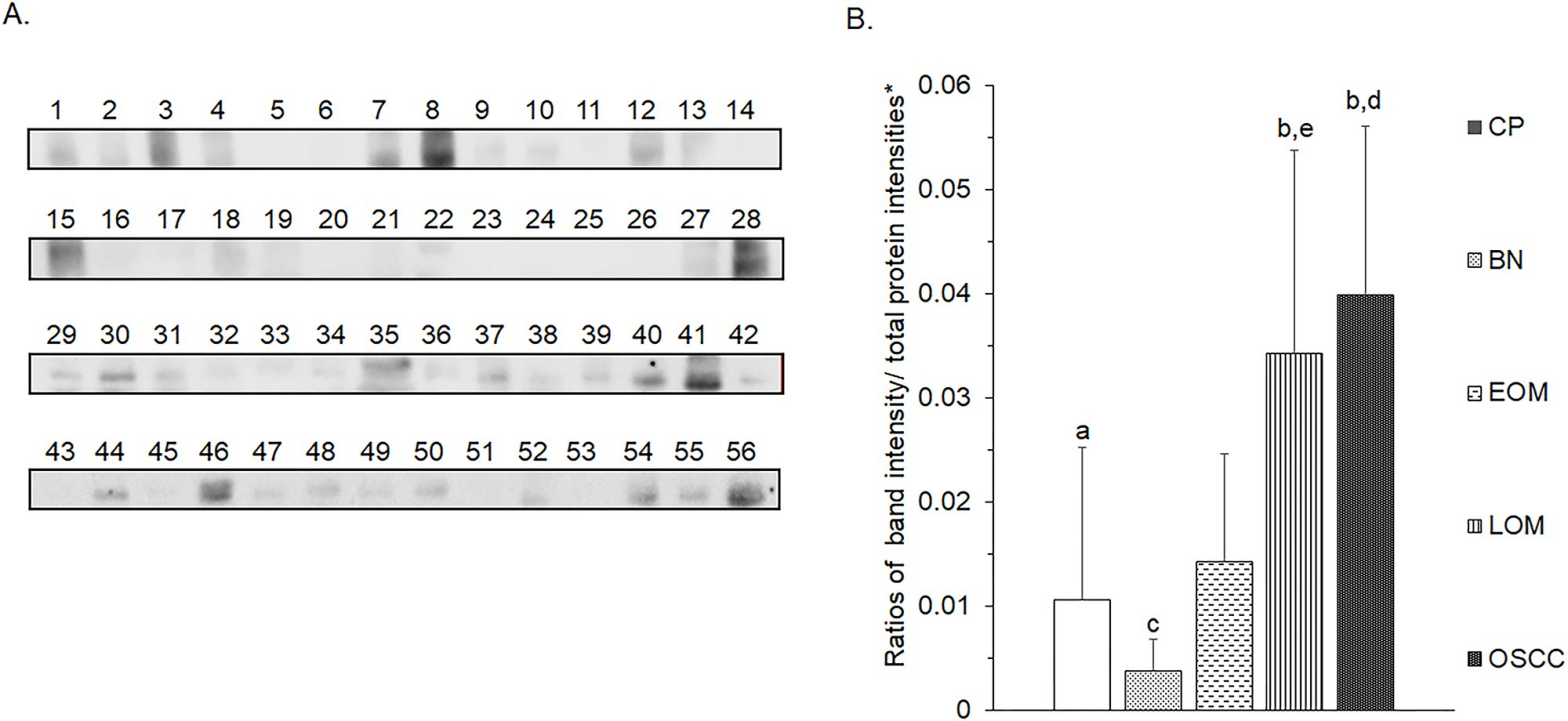
Western blot analysis of salivary toll like receptor 4 (TLR4) of individual saliva samples from dogs with benign oral tumors (BN), early- and late-stage oral melanoma (EOM and LOM, respectively), oral squamous cell carcinoma (OSCC) and periodontitis and normal controls (CP). Representative western blot for TLR4 at 95 kDa **(A)** and bar graph of ratios of TLR4 protein intensity to total blotted proteins in each lane in the first half of a membrane* **(B)**. a–b, c-d and c-e denote a significant difference at p<0.05, p<0.01 and p<0.001, respectively.

**Fig 14:**
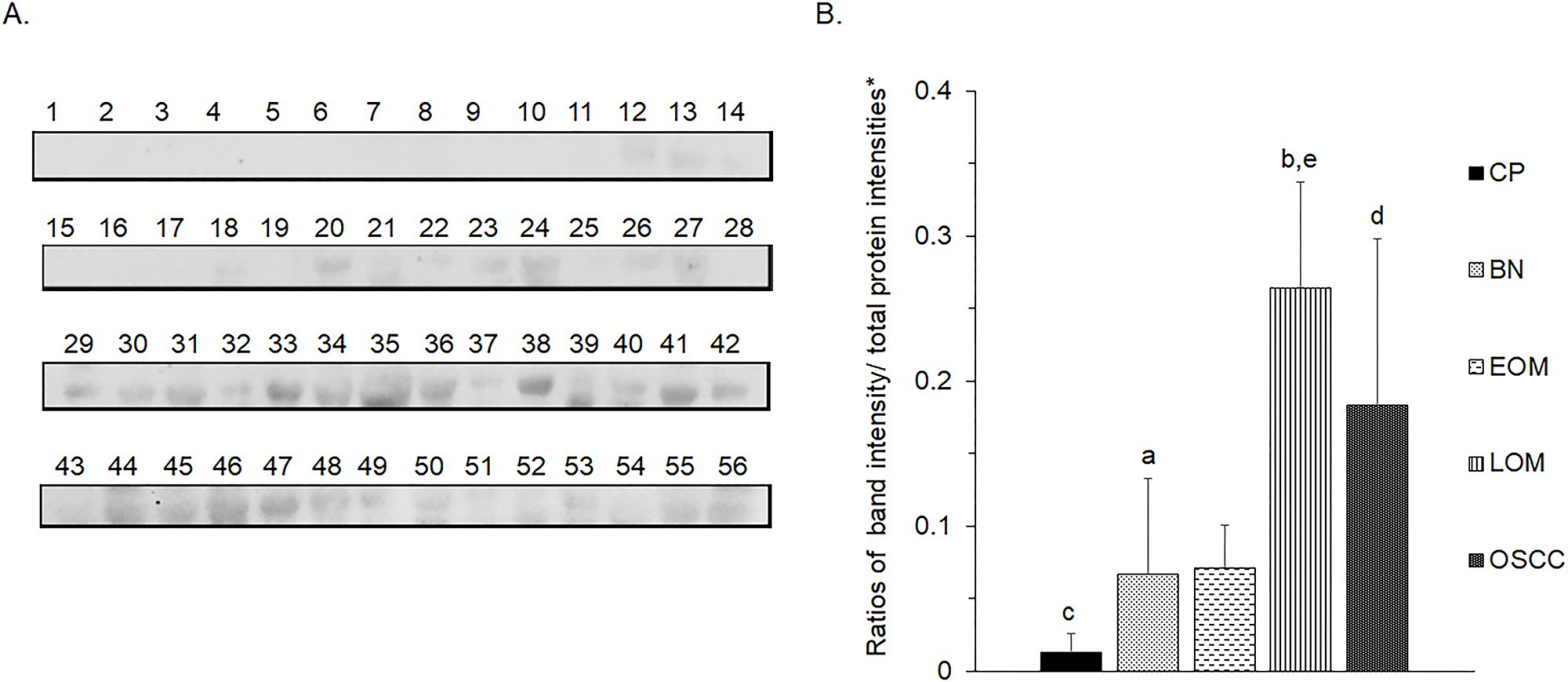
Western blot analysis of nuclear factor kappa B (NF-κB) of individual saliva samples from dogs with benign oral tumors (BN), early- and late-stage oral melanoma (EOM and LOM, respectively), oral squamous cell carcinoma (OSCC) and periodontitis and normal controls (CP). Representative western blot for NF-κB at 65 kDa **(A)** and bar graph of ratios of NF-κB protein intensity to total blotted proteins in each lane in the first half of a membrane* **(B)**. a–b, c-d and c-e denote a significant difference at p<0.05, p<0.01 and p<0.001, respectively.

All 32 replicates in each pooled sample group demonstrated the homogeneity within the group. A 3-dimensional view of the PCA plot showed distinct clusters among the EOM, LOM, OSCC and BN groups, whereas periodontitis and healthy controls were shown to be in the same cluster and classified as a control (CP) group (Fig 1). The MALDI-TOF MS results had an accurate outcome with the 95% confidence interval. The cross-validation, calculated by ANOVA, in the CP, BN, EOM, LOM, and OSCC was 100%, 100%, 96.88%, 100% and 100%, respectively, and the recognition capability, calculated by QC/ Different Average, SNN, AD, TTA, W/KW and the Genetic Algorithm (GA) test in the CP, BN, EOM, LOM, and OSCC groups was all 100%, indicating that the results were of high reliability. Divergent PMFs of CP, EOM, LOM, OSCC and BN groups were observed, and peptide masses at 1000–5000 Da were selected by ClinProTools software and specific peptide sequences were analyzed by LC MS/MS. Seven peptide fragments appeared in the tumor groups (EOM, LOM, OSCC and BN) at 1096, 1208, 1322, 1794, 1864, 2354 and 2483 Da (SENP7 or KAT2B, PPRC1 or RMND1, DTX3L, ZNF699, MAP3K15 or ATP6V1E2, PLCL2 and COL12A1, respectively), two peptide fragments appeared in the LOM and OSCC groups at 2450 and 3492 Da (TNRC18 and TLR4, respectively), two peptide fragments appeared only in the CP controls at 2544 and 3026 Da (ZNF451 and CASPL4A2, respectively) (Figs 2–4). Candidate protein biomarkers were evaluated for biological processes and location in the cell by UniProtKB/Swiss-Prot (Table 1) [26]. Networks of protein–protein and protein–chemotherapy drug interactions were performed by the Stitch program, version 5.0 and pathways with high edge confidence scores (>0.700) represented the strength of the protein–protein interactions at the functional level (Figs 5–7) [27]. Several candidate proteins presented in this study showed a strong relationship with chemotherapy drugs, including KAT2B, PPRC1, DTX3L, ZNF699 and MAP3K15. Also, p53 was noticeable in all of these pathways as well as the pathways of SENP7-doxorubicin except SENP7-cyclophosphamide/piroxicam and SLC30A10-cyclophosphamide/piroxicam pathways which involved the cytochrome P450 family 2 (CYP2) family. We did not find an association of TLR4 with chemotherapy drugs. However, western blot analysis revealed protein expression of SENP7 in EOM, LOM, OSCC and BN, and TLR4 and NF-κB in LOM and OSCC (Figs 8–10, S1–S3 Figs and S1 Table). The protein bands of SENP7, TLR4 and NF-κB on the membranes were verified by LC-MS/MS. From the Mascot search results, MS/MS fragmentations of KFRKTLPR, NLRYLDISYTR and MLLAVQR were found to be matched with SENP7, TLR4, and NF-κB, respectively (Fig 11). Western blots were then performed in individual samples. The results were shown in Figs 12–14 and S4–S6 Figs. The increased expression of SENP7, TLR4 and NF-κB was observed in LOM and OSCC compared with CP and BN.

**Table 1:** Nominated proteins based on biological process involvement and protein score, using MALDI-TOF MS and LC-MS/MS data.

Database	Protein name	Protein score	Peptide sequence	Biological process	Subcellular distribution
XP_010329708.1	Sentrin-specific protease 7 isoform X1 (SENP7)	29	LNLSERIPR	Adenylate cyclase-modulating G-protein-coupled receptor signaling pathway	Nucleus and plasma membrane
XP_021512678.1	Histone acetyltransferase KAT2B (KAT2B)	25	VYPGLLCFK	Cell cycle arrest, chromatin remodeling	Nucleus and cytoskeleton
EHB18528.1	Peroxisome proliferator-activated receptor gamma coactivator-related protein 1 (PPRC1)	17	AHDHYQRQR	Positive regulation of DNA-binding transcription factor activity	Nucleus
ERE86066.1	Required for meiotic nuclear division protein 1-like protein (RMND1)	9	TLALSTYFHR	Positive regulation of mitochondrial translation	Mitochondrion
A0A286Y4B2	E3 ubiquitin-protein ligase DTX3L (DTX3L)	15	TLYGIQTGNQPK	Histone ubiquitination	Cytosol, endosome, lysosome and nucleus
XP_012863361.1	Zinc finger protein 699 (ZNF699)	14	EYGEACSSPSSIGPPVR	Regulation of transcription	Nucleus
XP_004612329.1	Mitogen-activated protein kinase kinase kinase 15 (MAP3K15)	20	TDSMEILTSDIIDGLLK	Activation of MAPKK activity	Nucleus and cytoplasm
XP_021074063.1	V-type proton ATPase subunit E 2 (ATP6V1E2)	16	VCNTLESRLNLAAMQK	ATP hydrolysis coupled proton transport	Membrane
XP_010964328.1	Inactive phospholipase C-like protein 2 (PLCL2)	17	VMVMTSPNVEESYLPSPDVLK	Intracellular signal transduction	Cytoplasm
ELK17433.1	Trinucleotide repeat-containing protein 18 protein (TNRC18)	16	NSSGKLSGKPLLTSDAYELGAGMR	Chromatin silencing	Cytosol, mitochondrion, nucleus and other locations
XP_007951446.1	Collagen type XII alpha-1 chain (COL12A1)	23	DYKPQVGVIVDPSTKTLSFFNK	Cell adhesion	Extracellular matrix
XP_004267830.1	Zinc finger protein 451 isoform X2 (ZNF451)	18	DTSPFQPNPPAGGPIVEALEHSKR	Nucleic acid binding	Nucleus
XP_015104668.1	Protocadherin Fat 1 isoform X3 (FAT1)	16	GNPPMSEITSVHIFVTIADNASPKFTSK	Homophilic cell adhesion via plasma membrane adhesion molecules	Plasma membrane and integral component of membrane
XP_016818046.1	CASP-like protein 4A2 (CASPL4A2)	22	SAASPGPAPAAGDPGGSARPRPAAPLGSALALAF	Iron–sulfur cluster binding	Plasma membrane
XP_003924923.1	Centrosomal protein of 192 kDa isoform X1 (CEP192)	21	SGNLLETHEVDLTSNSEELDPIRLALLGK	Centrosome-templated microtubule nucleation	Cytoskeleton
ERE87034.1	Glypican-5 (GPC5)	13	GMCKDLTKPMQHHVTVIAASTECVVTLK	Regulation of signal transduction	Plasma membrane and extracellular space
XP_010635607.1	Cell-cycle checkpoint protein RAD17 isoform X2 (RAD17)	13	LLFPKEIQEECSILNISFNPVAPTIMMK	Cell cycle	Nucleus
ABU41662.1	Toll-like receptor 4 variant 1 (TLR4)	3	MMSASRLAGTLIPAMAFLSCVRPESWEPCVE	Activation of MAPK activity	Early endosome and cell membrane

## Discussion

This study demonstrated the different salivary PMFs and clusters of EOM, LOM, OSCC, BN and CP groups by MALDI-TOF MS. In addition, unique protein expressions were observed by LC MS/MS and verified by western blotting. According to the discrete sample groups from the PCA, MALDI-TOF MS can possibly be used as a rapid and reliable method for detection of canine oral tumors. MALDI-TOF MS has been reported to be a potential tool to characterize human head and neck squamous cell carcinoma from oral brush biopsy, human OSCC from oral fluid and canine oral tumors from tissues [29–31]. Specific mass signals were found from saliva of oral lichen planus (OLP) patients as well as the discovery of a relationship between OLP and oral cancer [8]. Moreover, salivary PMFs showed a number of proteins that were differently expressed in the early-stage OSCC compared with healthy patient, using MALDI-TOF MS combined with magnetic beads [32]. The fast, high accuracy and high sensitivity of MALDI-TOF MS made it suitable for screening oral cancers from biological fluids, especially saliva, which was easy to collect. This could help reduce the recurrence of the disease in the future. However, more data from individual patients are required to set databanks of PMFs and PCA plots of the diseases.

Mass spectral peaks were analyzed for peptide and nominated protein identification. Differential protein expression has been revealed in canine oral tumors by MALDI-TOF MS coupled with LC-MS/MS and by in-gel digestion coupled with mass spectrometry (GeLC MS/MS) from tissues of oral tumors [33]. We did not find similar proteins to those from tumor tissues which was probably due to the different source of samples, as proteins in saliva could be either secretary proteins from zygomatic, parotid, mandibular and sublingual salivary glands or proteins from oral tumors. Compared with the previously reported normal canine salivary proteomics, a number of different proteins in the CP group were observed [12–15]. This was possibly owing to different groups of dogs in the study as we combined periodontitis and healthy dogs as a CP group. In addition, different dog breeds and environments as well as different proteomic approaches could affect the results [13–15].

Our study showed for the first time elevated SENP7 in canine oral tumors. SENPs are small ubiquitin-like modifier (SUMO)-specific protease. SENP7 functioned to deconjugate SUMO from cellular substrates [34]. SENP7 is in the same family as SENP3. Overexpressed SENP3 leads to the imbalance of SUMO homeostasis and to development and progression of a number of cancers including prostate, ovarian, lung, rectum, and colon [34]. The long SENP7 transcript has been reported to promote epithelial–mesenchymal transition and decrease the cell adhesion molecule E-cadherin (CDH1) in breast cancer cell line, which could lead to the metastasis of the disease [35]. Decreased mRNA expression of cell adhesion molecules [*CDH1*, syndecan-1 (*SDC1*) and *NECTIN4*] has been reported in canine OM [36]. SENP7 also showed a strong relationship with the chemotherapy drugs (Fig 5A–5C). The networks of SENP7 as well as other targets, KAT2B and DTX3L, with chemotherapy drugs showed the strong relationship with TP53 protein, a biomarker of oral cancers [(Figs 5 and 6) [37, 38]. The association of target proteins with TP53 and chemotherapy drugs, especially carboplatin and doxorubicin, should be further investigated.

TLR4 expression has been reported in several cancers, including human head and neck cancer [39], human laryngeal and oral cancer cell line and melanoma [40–42]. In fact, TLR4 has been reported to play divergent roles, either as a pro- or an anti-tumor agent [43]. Our study demonstrated that TLR4 and NF-κB expressions were elevated in LOM and OSCC, which displayed intimate clusters in a PCA plot; hence, TLR4 promoted cancer progression and possibly served as a prognostic factor. In addition, TLR4 has been reported to regulate the inflammatory response by activating NF-κB either via the myeloid differentiation primary response protein 88 (MyD88)-dependent pathway or the Toll/interleukin-1 receptor-domain-containing adapter-inducing interferon-β (TRIF)-dependent pathway [39, 44]. The TLR4–NF-κB pathway has been intensively studied in several cancers such as laryngeal carcinoma and ovarian carcinoma as prognostic markers and inhibiting of the pathway might serve as a potential treatment of the cancers. Suppressor of cytokine signaling 1 (SOCS1), a regulator of cytokine-mediated innate and adaptive immunity, has been reported to inhibit the TLR4–NF-κB pathway in laryngeal carcinoma. In addition, decreased TLR4 expression has been observed in ovarian carcinoma after treating with NF-κB inhibitor [45].

## Conclusions

The present study revealed the discrete clusters of EOM, LOM, OSCC, BN and CP groups, using salivary MALDI-TOF MS. With the combination of MALDI-TOF MS and LC MS/MS, potential protein candidates associated with the diseases were identified. Western blot analysis could verify SENP7, TLR4 and NF-κB as potential salivary biomarkers of canine oral tumors. Further studies of the role of TLR4 and NF-κB as prognostic markers of progressive tumors and of the signal transduction of SENP7 and TLR4–NF-κB pathways and the mechanisms to inhibit the pathways should be performed.

## Supporting information

S1 Fig Representative western blot of pooled samples for SENP7 at 37 kDa. Lane 1: Periodontitis and normal controls; Lane 2: Benign oral tumors; Lane 3: Early-stage oral melanoma; Lane 4: Late-stage oral melanoma; Lane 5: Oral squamous cell carcinoma.
(TIF)

S2 Fig Representative western blot of pooled samples for TLR4 at 95 kDa. Lane 1: Periodontitis and normal controls; Lane 2: Benign oral tumors; Lane 3: Early-stage oral melanoma; Lane 4: Late-stage oral melanoma; Lane 5: Oral squamous cell carcinoma.
(TIF)

S3 Fig Representative western blot of pooled samples for NF-κB at 65 kDa. Lane 1: Periodontitis and normal controls; Lane 2: Benign oral tumors; Lane 3: Early-stage oral melanoma; Lane 4: Late-stage oral melanoma; Lane 5: Oral squamous cell carcinoma.
(TIF)

S4 Fig Representative western blot of individual samples for SENP7 at 37 kDa. CTRL, 6 normal controls; PD, 3 Periodontitis; EOM, 5 Early-stage oral melanoma; BN, 11 Benign oral tumors; OSCC, 9 Oral squamous cell carcinoma; LOM, 22 Late-stage oral melanoma.
(TIF)

S5 Fig Representative western blot of individual samples for TLR4 at 95 kDa. CTRL, 6 normal controls; PD, 3 Periodontitis; EOM, 5 Early-stage oral melanoma; BN, 11 Benign oral tumors; OSCC, 9 Oral squamous cell carcinoma; LOM, 22 Late-stage oral melanoma.
(TIF)

S6 Fig Representative western blot of individual samples for NF-κB at 65 kDa. CTRL, 6 normal controls; PD, 3 Periodontitis; EOM, 5 Early-stage oral melanoma; BN, 11 Benign oral tumors; OSCC, 9 Oral squamous cell carcinoma; LOM, 22 Late-stage oral melanoma.
(TIF)

S1 Table The ratios of target band intensities from pooled samples to the total proteins in each lane in the first or second half of a membrane according to the sizes of target proteins. (XLSX)
