# Supplementary figures and images for: Salivary proteomics of canine oral tumors using MALDI-TOF mass spectrometry and LC-tandem mass spectrometry
Source: PLoS One. 2019 Jul 18;14(7):e0219390. doi: 10.1371/journal.pone.0219390 (PMC6638856; doi:10.1371/journal.pone.0219390)

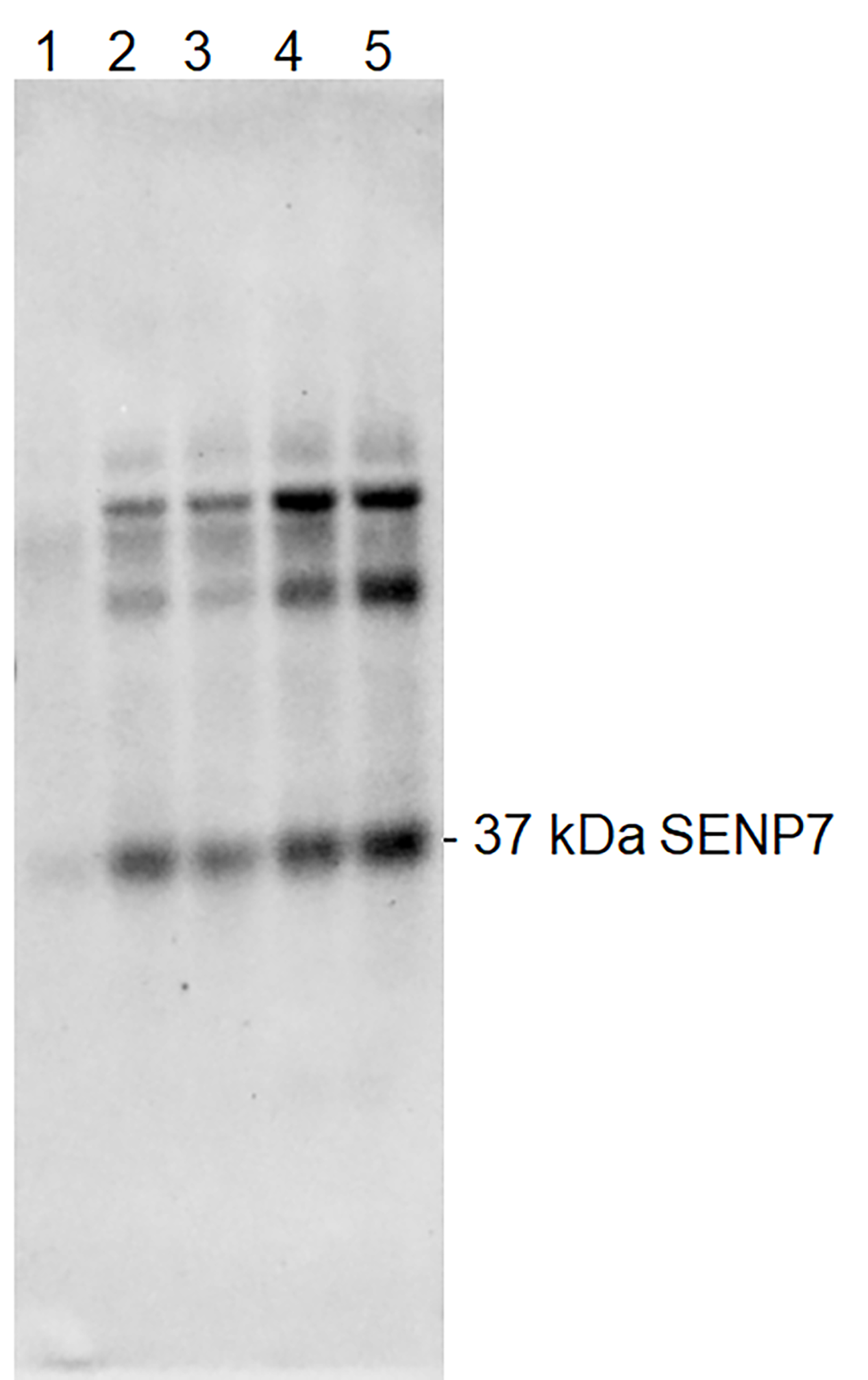

Supplement: S1 Fig — Lane 1: Periodontitis and normal controls; Lane 2: Benign oral tumors; Lane 3: Early-stage oral melanoma; Lane 4: Late-stage oral melanoma; Lane 5: Oral squamous cell carcinoma. (TIF) [file pone.0219390.s001.tif]

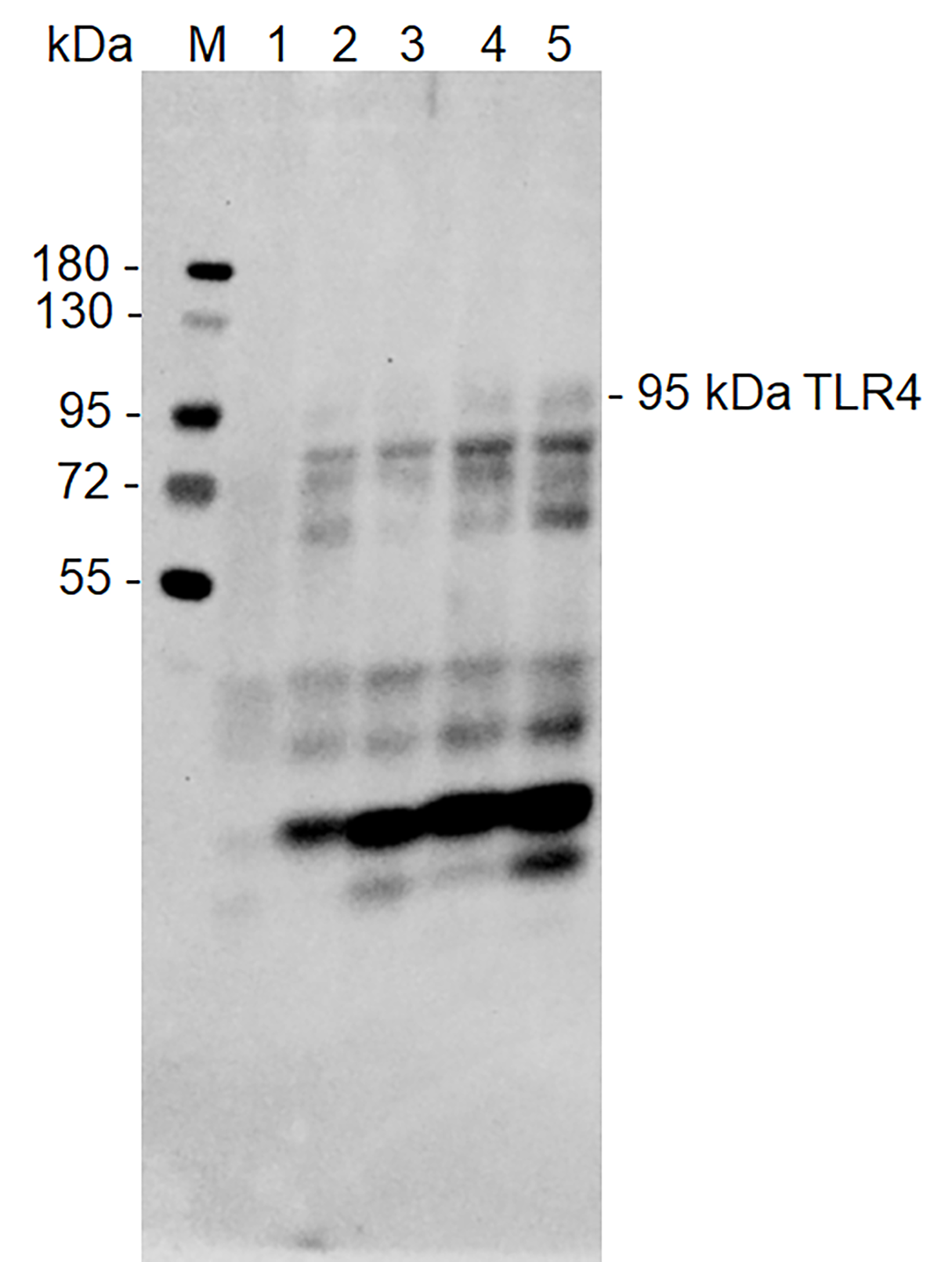

Supplement: S2 Fig — Lane 1: Periodontitis and normal controls; Lane 2: Benign oral tumors; Lane 3: Early-stage oral melanoma; Lane 4: Late-stage oral melanoma; Lane 5: Oral squamous cell carcinoma. (TIF) [file pone.0219390.s002.tif]

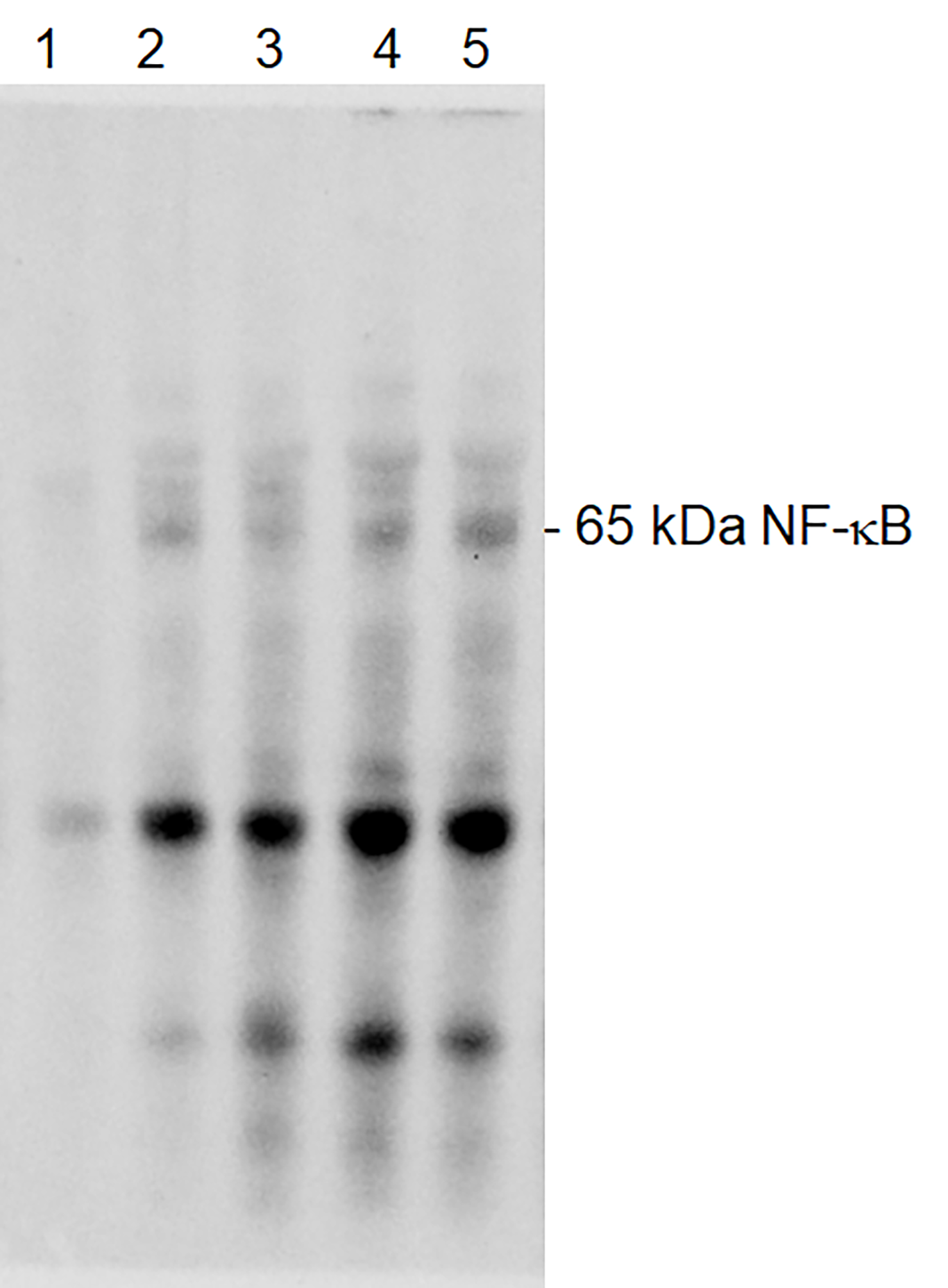

Supplement: S3 Fig — Lane 1: Periodontitis and normal controls; Lane 2: Benign oral tumors; Lane 3: Early-stage oral melanoma; Lane 4: Late-stage oral melanoma; Lane 5: Oral squamous cell carcinoma. (TIF) [file pone.0219390.s003.TIF]

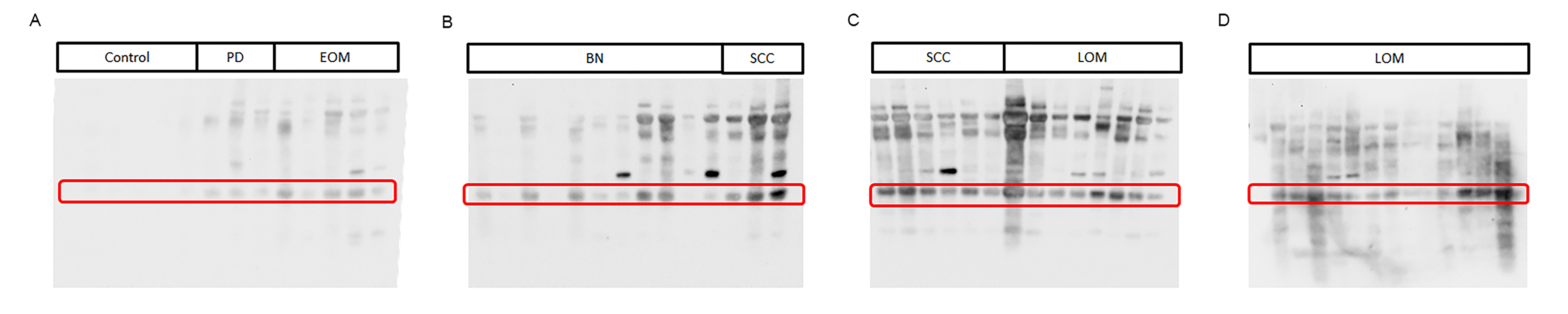

Supplement: S4 Fig — CTRL, 6 normal controls; PD, 3 Periodontitis; EOM, 5 Early-stage oral melanoma; BN, 11 Benign oral tumors; OSCC, 9 Oral squamous cell carcinoma; LOM, 22 Late-stage oral melanoma. (TIF) [file pone.0219390.s004.tif]

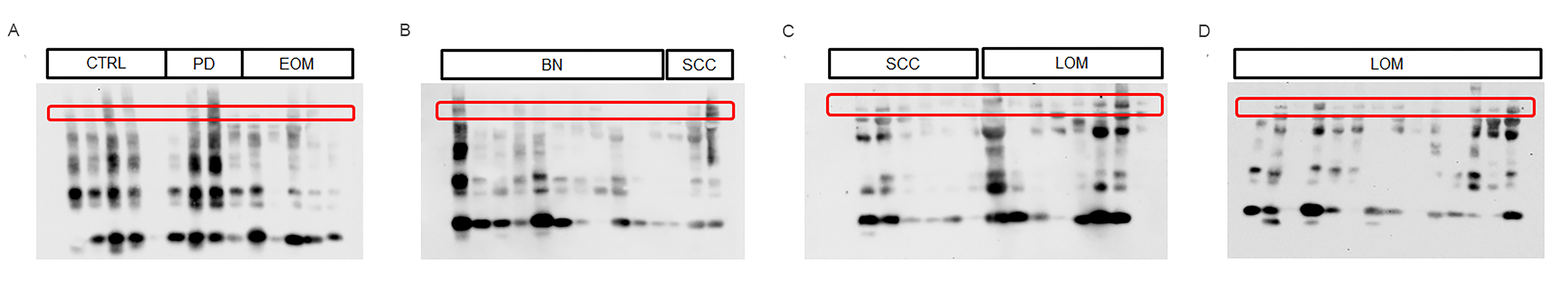

Supplement: S5 Fig — CTRL, 6 normal controls; PD, 3 Periodontitis; EOM, 5 Early-stage oral melanoma; BN, 11 Benign oral tumors; OSCC, 9 Oral squamous cell carcinoma; LOM, 22 Late-stage oral melanoma. (TIF) [file pone.0219390.s005.tif]

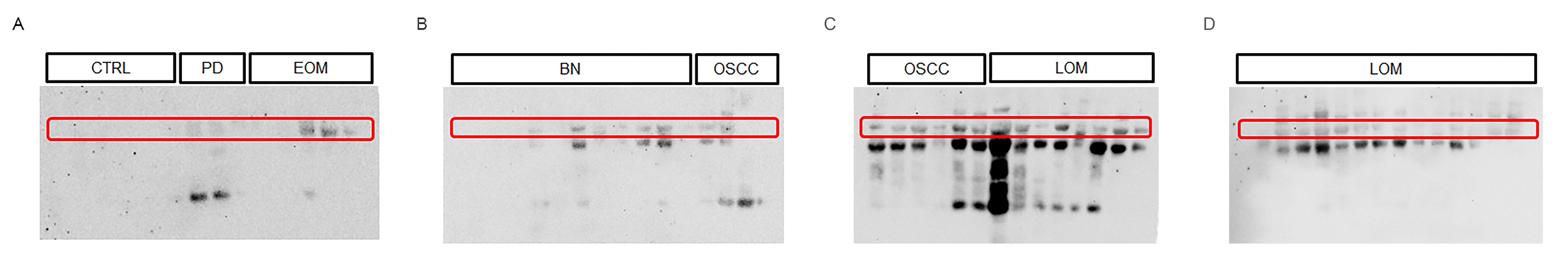

Supplement: S6 Fig — CTRL, 6 normal controls; PD, 3 Periodontitis; EOM, 5 Early-stage oral melanoma; BN, 11 Benign oral tumors; OSCC, 9 Oral squamous cell carcinoma; LOM, 22 Late-stage oral melanoma. (TIF) [file pone.0219390.s006.TIF]
